# Inhibition of transforming growth factor beta signaling pathway promotes differentiation of human induced pluripotent stem cell-derived brain microvascular endothelial-like cells

**DOI:** 10.1186/s12987-020-00197-1

**Published:** 2020-05-26

**Authors:** Misaki Yamashita, Hiromasa Aoki, Tadahiro Hashita, Takahiro Iwao, Tamihide Matsunaga

**Affiliations:** Department of Clinical Pharmacy, Graduate School of Pharmaceutical Sciences, Nagoya City University, 3-1 Tanabe-dori, Mizuho-ku, Nagoya, 467-8603 Japan

**Keywords:** Blood–brain barrier, Cell differentiation, Endothelial cells, Induced pluripotent stem cells, Transforming growth factor beta

## Abstract

**Background:**

The blood–brain barrier (BBB) plays an important role as a biological barrier by regulating molecular transport between circulating blood and the brain parenchyma. In drug development, the accurate evaluation of BBB permeability is essential to predict not only the efficacy but also the safety of drugs. Recently, brain microvascular endothelial-like cells derived from human induced pluripotent stem cells (iPSCs) have attracted much attention. However, the differentiation protocol has not been optimized, and the enhancement of iPSC-derived brain microvascular endothelial-like cells (iBMELCs) function is required to develop highly functional BBB models for pharmaceutical research. Thus, we attempted to improve the functions of differentiated iBMELCs and develop a versatile BBB model by modulating TGF-β signaling pathway without implementing complex techniques such as co-culture systems.

**Methods:**

iPSCs were differentiated into iBMELCs, and TGF-β inhibitor was used in the late stage of differentiation. To investigate the effect of TGF-β on freezing–thawing, iBMELCs were frozen for 60–90 min or 1 month. The barrier integrity of iBMELCs was evaluated by transendothelial electrical resistance (TEER) values and permeability of Lucifer yellow. Characterization of iBMELCs was conducted by RT-qPCR, immunofluorescence analysis, vascular tube formation assay, and acetylated LDL uptake assay. Functions of efflux transporters were defined by intracellular accumulation of the substrates.

**Results:**

When we added a TGF-β inhibitor during iBMELCs differentiation, expression of the vascular endothelial cell marker was increased and blood vessel-like structure formation was enhanced. Furthermore, TEER values were remarkably increased in three iPSC lines. Additionally, it was revealed that TGF-β pathway inhibition suppressed the damage caused by the freezing–thawing of iBMELCs.

**Conclusion:**

We succeeded in significantly enhancing the function and endothelial characteristics of iBMELCs by adding a small molecular compound, a TGF-β inhibitor. Moreover, the iBMELCs could maintain high barrier function even after freezing–thawing. Taken together, these results suggest that TGF-β pathway inhibition may be useful for developing iPSC-derived in vitro BBB models for further pharmaceutical research.

## Background

The blood–brain barrier (BBB) plays a very important biological barrier role by regulating molecular transport between the circulating blood and the brain parenchyma [1]. The BBB consists of brain microvascular endothelial cells (BMECs), which are characterized by specialized tight junctions [2, 3] and high expression of multidrug efflux transporters [4].

In drug development, accurate evaluation of BBB permeability is required to predict not only the efficacy but also the safety of drugs [5]. To evaluate BBB permeability, in vitro models are often used; however, there is no ideal in vitro BBB model available currently. Although BBB permeability has been evaluated by primary BMECs obtained from animals [6–8], species differences between human and animals have made it difficult to apply accurately animal findings in humans [9]. In addition, it is very difficult to obtain primary human BMECs for pharmacokinetic studies. Human immortalized BMECs have the advantage of promoting reproducibility of experimental data. However, it is difficult to predict BBB permeability accurately due to low barrier functions in most human immortalized BMECs [10] compared with physiological transendothelial electrical resistance (TEER) values. The reported physiological TEER values are more than 1000 Ω × cm^2^ in capillaries of rat or frog brain [11, 12]. Thus, non-endothelial cells with high-TEER values, such as Caco-2 cells and Madin–Darby canine kidney cells, have been used for pharmacokinetic studies [13]. However, the functions of transporters in these cell lines differ from those in human BMECs. Therefore, there is a focus on generating functional BMECs from human induced pluripotent stem cells (iPSCs), which, because of pluripotency, can differentiate into various cell types in our organs and tissues [14].

Recently, some human iPSC-derived BBB models have been developed [15–23]. However, expression levels of endothelial cell markers were low [18], and the barrier functions were unstable depending on the iPSC line used [17, 23]. Therefore, these reports suggested that the differentiation protocol has not been optimized and enhancement of iPSC-derived brain microvascular endothelial-like cells (iBMELCs) function is required to develop highly functional BBB models for pharmaceutical research. Previously, co-culture systems with pericytes, astrocytes, or neurons—all components of BBB—have been tried to promote differentiation into highly functional iBMELCs [15–21]. However, the systems are not reproducible and suitable for drug screening due to complex manipulation. Additionally, although cryopreservation of iBMELCs using Rho-associated coiled-coil forming kinase inhibitor Y-27632 has been reported, iBMELCs were not able to maintain the barrier function for a prolonged period after freezing–thawing [24]. Thus, we attempted to improve the functions of differentiated iBMELCs and develop a versatile BBB model without implementing complex techniques such as co-culture systems.

Transforming growth factor beta (TGF-β) signaling is involved in embryonic development including cell differentiation, apoptosis, cellular homeostasis, and other cellular functions [25, 26]. Furthermore, TGF-β is associated with BBB dysfunction [27–29]. However, the effect of TGF-β inhibition on iBMELCs differentiation has not be analyzed. In this study, we show that TGF-β inhibitors critically enhance the BBB properties of human iBMELCs without complex manipulations such as the co-culture with pericytes or astrocytes. This approach may provide a powerful tool in pharmaceutical research.

## Methods

### Culture of human iPSCs

Human iPSCs (610B1, 606A1 and 648A1) were purchased from *RIKEN* (Saitama, Japan) and were maintained on a feeder layer of mitomycin C-treated mouse embryonic fibroblasts in iPSC medium [Dulbecco’s Modified Eagle’s Medium/Ham’s F12 (Wako Pure Chemical Industries (Wako), Osaka, Japan) containing 20% KnockOut Serum Replacement (Invitrogen, Carlsbad, CA, USA), 2 mM l-glutamine (Wako), 1% minimal essential medium with non-essential amino acids (Invitrogen), 0.1 mM β-mercaptoethanol (Sigma-Aldrich, St. Louis, MO, USA), and 5 ng/mL human fibroblast growth factor-2 (FGF-2) (GenScript, Nanjing, China)] at 37 °C in 5% CO_2_.

### Differentiation of human iPSCs into BMELCs

**Fig. 1:**
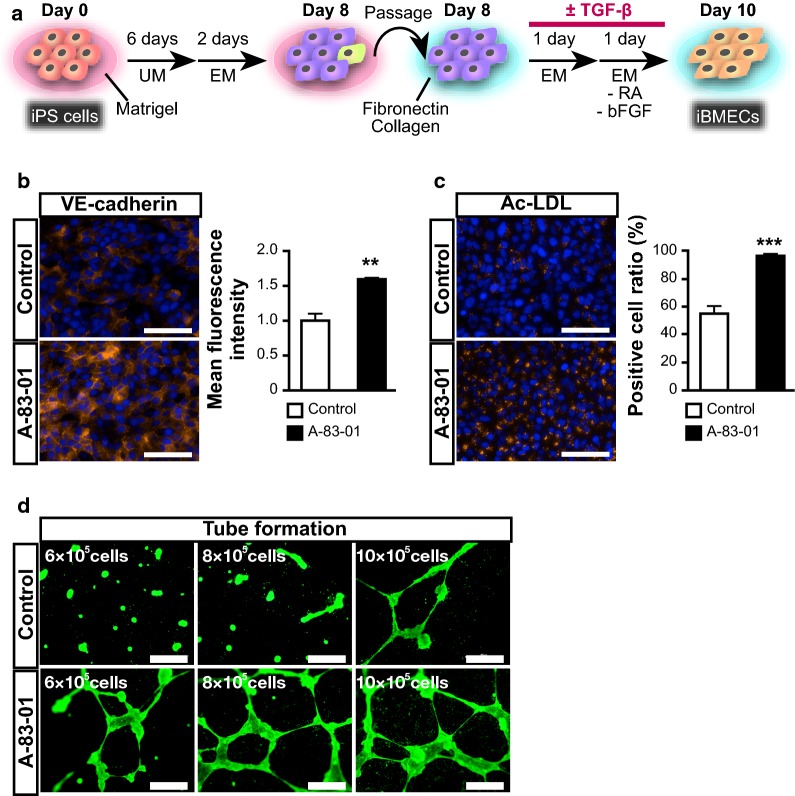
The effect of TGF-β inhibitor on iBMELCs differentiation. **a** A schematic diagram of the protocol of differentiation of human iPSCs to BMECs. **b** Immunofluorescence for the endothelial cell adhesion molecule (VE-cadherin: red). Blue: DAPI. Scale bar, 100 μm. Statistical significance was calculated using the unpaired Student’s *t*-test (**p < 0.01), control = 1. Data are represented as the mean ± SD (*n* = 3). **c** Ac-LDL (red) uptake assay. Blue: Hoechst 33342. Scale bar, 100 μm. Positive cell ratio was defined as the number of cells with Ac-LDL uptake to the total number of cells. Statistical significance was calculated using the unpaired Student’s *t*-test (***p < 0.001). Data are represented as the mean ± SD (*n* = 3). **d** Tube formation assay. The cells were stained with calcein (green). Scale bar, 500 μm

Prior to differentiation, human iPSCs were seeded onto Growth Factor Reduced Matrigel (Matrigel) (Corning, Corning, NY, USA)-coated plates and cultured with StemSure hPSC medium (Wako) supplemented with 35 ng/mL FGF2 for 3–4 days. Differentiation into human iPSC-derived BMECs was performed as previously described [15, 16]. The protocol has been described in Fig. 1a. Briefly, after reaching 70% confluence, cells were cultured in standard unconditioned medium (UM; iPSC medium without FGF2) for 6 days. The medium was changed every day. Then, the culture medium was switched to EC medium [Human Endothelial-SFM (Thermo Fisher Scientific, Waltham, MA, USA) supplemented with 1% platelet-poor plasma derived bovine serum (PDS) (Alfa Aesar, Haverhill, MA, USA), 20 ng/mL FGF2, and 10 μM all-*trans* retinoic acid (RA) (Tocris Bioscience, Bristol, UK)]. After 2 days, the cells were detached using Accutase (Nacalai Tesque, Kyoto, Japan) (20 min, 37 °C) and plated onto tissue culture polystyrene plates or 0.3-cm^2^ Transwell-Clear permeable inserts (0.4 μm pore size, Corning) coated with a mixture of fibronectin (100 μg/mL; Wako) and collagen IV (400 μg/mL; Nitta geratin, Osaka, Japan). The cells were seeded at a density of 3.0 × 10^5^ cells/insert and cultured for 24 h with EC medium. Thereafter, culture medium was replaced with EC medium lacking FGF2 and RA for 24 h. The cells were treated with 1 μM TGF-β inhibitors, A-83-01 (Wako), SB-431542 (Wako), and RepSox (Wako), from day 8 to day 10. As shown in Additional file 1: Fig. S5, the cells were treated with A-83-01 from day 8 to day 10, from day 8 to day 12, or from day 10 to day 12.

### Quantitative reverse transcription-polymerase chain reaction (RT-qPCR)

Total RNA was extracted using the Agencourt RNAdvance Tissue Kit (Beckman Coulter, Brea, CA, USA) according to the manufacturer’s instructions. cDNA was synthesized from 0.5 ng total RNA using ReverTra Ace qPCR RT Master Mix (Toyobo, Osaka, Japan). KAPA SYBR FAST qPCR Kit Master mix (2×) ABI Prism (Kapa Biosystems, Wilmington, MA, USA) was used as the reaction solution for RT-qPCR. The samples were amplified using a LightCycler 96 System (Roche Diagnostics, Basel, Switzerland). PCR primer sequences are shown in Additional file 1: Table S1. All values were normalized by the value of the hypoxanthine phosphoribosyltransferase (HPRT) gene.

### Immunofluorescence staining

Cells were differentiated after seeding on Cell carrier-96 (PerkinElmer, Waltham, MA, USA) on day 8. On day 10, differentiated cells were washed with phosphate buffered saline (PBS) and fixed for 20 min in 4% paraformaldehyde. Next, the cells were washed three times with PBS containing 10 mM glycine and then permeabilized in PBS containing 0.1% Triton X-100 for 25 min. After blocking with 5% donkey serum for 20 min, cells were incubated with the primary antibody for 120 min, followed by incubation with both secondary antibody and a 1:1000 dilution of 4′,6-diamidino-2-phenylindole (DAPI) (Dojindo, Kumamoto, Japan) for 60 min. The cells were then washed three times with PBS. The list of antibodies used in this study is shown in Additional file 1: Table S2.

For vascular endothelial cadherin (VE-cadherin), von Willebrand factor (vWF), claudin-5, P-glycoprotein (P-gp), breast cancer resistant protein (BCRP), and glucose transporter 1 (GLUT1) staining, differentiated cells were washed three times with PBS containing 0.1% bovine serum albumin (BSA), fixed with 4% paraformaldehyde for 15 min, washed three times with PBS containing 0.1% BSA, and permeabilized with 0.1% Triton X-100 for 5 min. After washing three times with PBS containing 0.1% BSA, cells were incubated overnight at 4 °C with primary antibodies diluted in PBS containing 0.1% BSA. The cells were washed three times with PBS containing 0.1% BSA and incubated with a 1:200 dilution of Alexa Fluro 564-labeled secondary antibody for 60 min. After washing three times with PBS containing 0.1% BSA, the cells were incubated with a 1:1000 dilution of DAPI for 5 min. The cells were subsequently treated with 4% paraformaldehyde for 5 min and washed three times with PBS.

Immunofluorescence images were observed using the Operetta High-Content Imaging System (PerkinElmer). Positive cells were counted using the Harmony software, and the average number of cells from three wells (6 fields/well) was considered. Positive cell ratio was calculated by dividing the number of positive cells by the total cell number. Fluorescence intensity was also calculated using the Harmony software as the average fluorescence intensity from three wells (6 fields/well). The mean fluorescence intensity was calculated by dividing the fluorescence intensity by the total cell number. The settings used for the Opperetta High-Content Imaging System are shown in Additional file 1: Table S3.

### Vascular tube formation assay

Differentiated cells at day 8 were seeded onto 6-well plates pre-coated with a mixture of 100 μg/mL fibronectin and 400 μg/mL collagen IV. At day 10, the cells were plated onto 24-well plates pre-coated with 300 μL Matrigel and incubated in Human Endothelial-SFM supplemented with 1% PDS and 40 ng/mL VEGF, for 20–24 h. The cells were then treated with Calcein-AM for 30 min, and tube formation was observed using an ECLIPSE Ni microscope (NIKON, Tokyo, Japan).

### Acetylated LDL uptake assay

iBMELCs were incubated with 10 μg/mL Ac-LDL (Alfa Aesar) for 5 h and then incubated for 30 min with 10 μg/mL Hoechst 33342. After washing four times with the medium, cells were analyzed using the Operetta High-Content Imaging System (PerkinElmer). Ac-LDL-positive cells were counted using the Operetta High-Content Imaging System as the average number in 6 fields/well in three wells. Positive cell ratio was calculated by dividing the number of positive cells by the total cell number. The settings used for the Opperetta High-Content Imaging System are shown in Additional file 1: Table S3.

### Western blot analysis

The differentiated cells were lysed in 1 × sodium dodecyl sulfate–polyacrylamide gel electrophoresis (SDS-PAGE) sample buffer. The protein samples were separated by SDS-PAGE, transferred to polyvinylidene fluoride membranes, which were blocked with 4% Block-Ace solution, washed with tris-buffered saline containing 0.1% Tween 20 (TBS-T), incubated with primary antibodies (Additional file 1: Table S3) at room temperature for 1 h followed by incubation with secondary antibodies at room temperature for 30 min (Additional file 1: Table S4). After washing with TBS-T, protein bands were detected using an Amersham Imager 600 system (GE Healthcare Life Sciences, Chicago, IL, USA).

### Paracellular permeability assay

iBMELCs were cultured on a Transwell culture insert. On day 10, the medium was replaced with transport buffer (Hanks’ balanced salt solution containing 10 mM HEPES), and the cells were cultured for 20 min at 37 °C. Transport buffer containing 300 μM Lucifer Yellow was added to the apical side, and transport buffer alone was added to the basolateral side. After 60 min of incubation at 37 °C, 100 μL of the solution was collected from the basolateral side. The volumes of transport buffer in the apical and basal sides were 300 and 800 μL, respectively. Fluorescent signals of LY were measured using the Synergy HTX multimode plate reader and analyzed using the Gen 5 data analysis software (BioTek Instruments, Inc., Winooski, VA, USA).

### Transport activity study

Differentiated cells at day 8 were seeded onto 24-well plates pre-coated with a mixture of 100 μg/mL fibronectin and 400 μg/mL collagen IV. At day 10, the culture medium was removed, and the cells were pre-incubated with the transport buffer at 37 °C for 15 min. Uptake was initiated by replacement with transport buffer containing 10 μM rhodamine 123 or 20 μM Hoechst 33342 at 37 °C in the presence or absence of 10 μM Ciclosporin A or 20 μM Ko 143, which are the inhibitors of P-gp or BCRP, respectively. After 60 min, the cells were washed three times with PBS and lysed using PBS with 5% Triton X-100 solution. Fluorescence intensity in the cells was measured using the Synergy HTX multimode plate reader and analyzed using the Gen 5 data analysis software (Bio-Tek, Winooski, VA, USA). The total protein of the differentiated cells was measured using Pierce™ BCA Protein Assay Kit (Thermo Fisher Scientific).

### TEER measurement

**Fig. 2:**
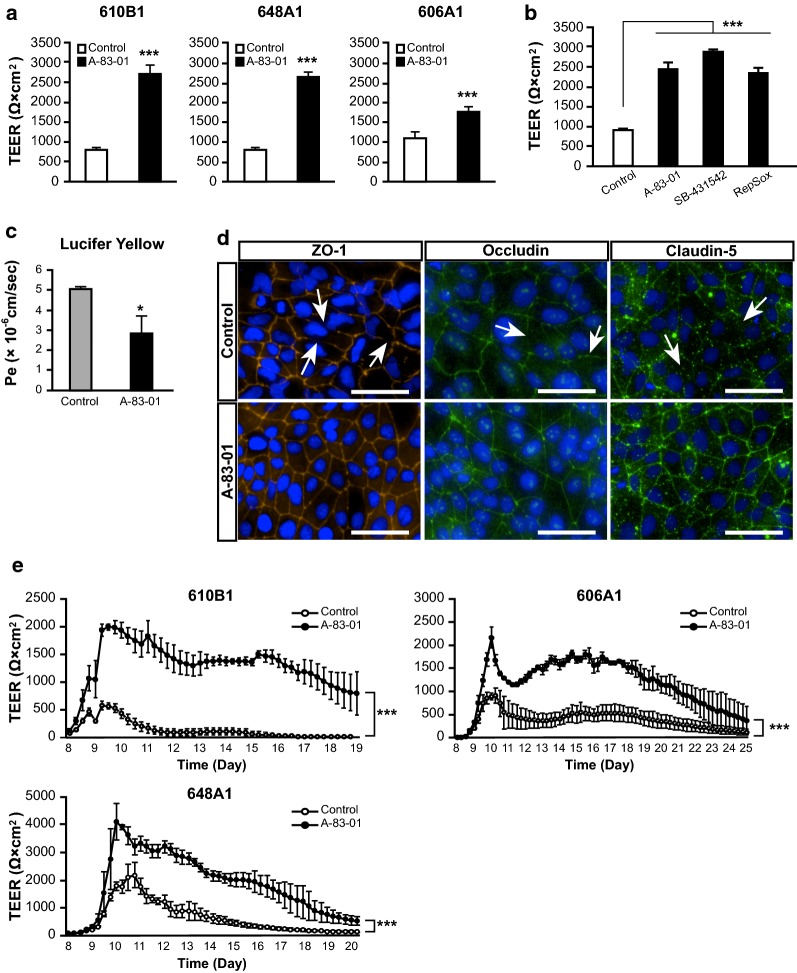
Tight junction analysis. **a** Effects of A-83-01 on the TEER values of BMECs derived from multiple iPSC lines. Statistical significance was calculated using the unpaired Student’s *t*-test (***p < 0.001). Data are represented as the mean ± SD (610B1, *n* = 3; 648A1, 606A1 *n* = 4). **b** Effects of TGF-β inhibitors (A-83-01, SB-431542, and RepSox) on the TEER values of BMECs. Statistical significance was calculated using one-way ANOVA and Tukey test (***p < 0.001), vs. control. Data are represented as the mean ± SD (610B1, *n* = 3). **c** Lucifer Yellow permeability coefficient (Pe) in iBMELCs. Statistical significance was calculated using the unpaired Student’s *t*-test (*p < 0.05). Data are represented as the mean ± SD (*n* = 3). **d** Immunofluorescence for the tight junction markers (ZO-1, occludin, and claudin-5). Blue: DAPI. White arrows: fragile and discontinuous tight junctions. Scale bar, 50 μm. **e** Time-course of the TEER values of BMECs derived from multiple iPSC lines. Statistical significance was calculated using two-way repeated measures ANOVA (***p < 0.001). Data are represented as the mean ± SD (648A1, *n* = 6; 610B1, 606A1, *n* = 4)

TEER values were measured using Millicell-ERS (Merck Millipore, Burlington, MA, USA) or cellZscope (CellSeed, Tokyo, Japan) according to the corresponding manufacturer’s instructions. TEER values shown in Fig. 2e were measured using cellZscope, whereas the others were measured using Millicell-ERS. During the measurement of the time-course of TEER values, the medium was switched to EC medium lacking FGF2, RA, and TGF-β inhibitor at day 10, and the medium was not changed thereafter. In this study, we defined values ≥ 1000 Ω × cm^2^ as high-TEER values.

### Freezing–thawing of iBMELCs

At day 8, the cells were incubated with Accutase for 20 min at 37 °C after washing with PBS. The cells were harvested with Human Endothelial-SFM and centrifuged at 100×*g* for 5 min. The cell pellets were resuspended with TC-protector (KAC, Kyoto, Japan) and frozen at − 80 °C. After 60–90 min (Fig. 4) or 1 month (Additional file 1: Fig. S9), frozen cells were quickly thawed with warm Human Endothelial-SFM. To remove the cell preservation solution, cells were transferred into 50 mL tubes and centrifuged at 100×*g* for 5 min. The cells were then resuspended with 1 mL of warm EC medium and seeded onto tissue culture polystyrene plates or 0.3-cm^2^ Transwell-Clear permeable inserts coated with a mixture of fibronectin and collagen IV. For subsequent operations, the procedure was the same as the above standard protocol on day 9 and later.

### Statistical analysis

The statistical significance of the differences between the two groups was assessed using the unpaired Student’s *t*-test. The statistical significance in Fig. 2b was assessed using one-way factorial analysis of variance (ANOVA) and Tukey’s HSD post hoc test for multiple comparisons. The statistical significance in Figs. 2e, 4b, Additional file 1: Figs. S4, S5 was assessed using two-way repeated measures ANOVA for multiple comparisons. SPSS statistics version 25.0 (IBM Japan, Tokyo, Japan) was used for statistical analyses. A *p*-value< 0.05 (two-tailed) indicates a statistically significant difference.

## Results

### The characteristics of TGF-β inhibitor-differentiated iBMELCs

We differentiated iBMELCs using the protocol described in Fig. 1a. To investigate the characteristics of iBMELCs as vascular endothelial cells, we performed immunofluorescence with endothelial markers, the acetylated-low density lipoprotein (Ac-LDL) uptake assay [30], and the tube formation assay. Expression of the vascular endothelial cell marker, VE-cadherin, was significantly increased in the A-83-01-treated group compared to controls (Fig. 1b, Additional file 1: Fig. S1a). Expression of the typical endothelial cell marker vWF was significantly increased in the A-83-01-treated group compared with that in controls, whereas the expression of the platelet endothelial cell adhesion molecule-1 (PECAM1) in the A-83-01-treated group was equivalent to that in controls (Additional file 1: Fig. S1b). Positive cell ratio of Ac-LDL uptake was also significantly increased in the A-83-01-treated group compared to controls (Fig. 1c, Additional file 1: Fig. S1c). Although approximately 10 × 10^5^ cells/well were needed to form a blood vessel-like structure in the control group, less than 10 × 10^5^ cells/well were sufficient for this formation in the A-83-01-treated group (Fig. 1d). TGF-β is known to mediate endothelial to mesenchymal transition (EndoMT) [31]. Thus, we hypothesized that EndoMT related to the enhancement of endothelial characteristics, and confirmed the expression level of the mesenchymal cell marker, neural cadherin (N-cadherin). As a result, the expression level of N-cadherin was significantly decreased when A-83-01 was added, in agreement with our hypothesis (Additional file 1: Fig. S2). To assess the activation of TGF-β signaling pathway using a standard protocol (control group) during differentiation, we attempted to detect the phosphorylation of SMAD2 by Western blotting analysis. We confirmed that the phosphorylated SMAD2 was detected in iBMELCs on days 8 and 10 (control group), whereas it was almost undetectable in A-83-01-treated iBMELCs on day 10 (Additional file 1: Fig. S3).

### The effects of TGF-β inhibitors on tight junction formation

TEER values, an index of the intensity of tight junctions, were measured in three different iPSC line-derived iBMELCs at day 10. In all cell lines, A-83-01 significantly increased the TEER value of iBMELCs (Fig. 2a). Similar effects were observed when using two other TGF-β inhibitors, SB-431542 and RepSox (Fig. 2b). iBMELC paracellular permeability (Pe) for Lucifer Yellow was also significantly lower in A-83-01-treated iBMELCs than in control (Fig. 2c). To examine the effect of TGF-β inhibitor on relatively mature endothelial cells, immortalized human BMEC, hCMEC/D3, and human umbilical vein endothelial cells (HUVEC) were treated with A-83-01. However, A-83-01 had little effect on the TEER values of hCMEC/D3 and HUVEC (Additional file 1: Fig. S4). The effects of TGF-β inhibitor addition on TEER values before and after the end of differentiation were investigated. Although the TEER values in days 10–12 A-83-01-treated group were significantly higher than those in the non-treated group, TEER values in days 8–10 A-83-01-treated and days 8–12 A-83-01-treated groups were not significantly different. The two groups treated with A-83-01 from days 8 to 10 (days 8–10-treated group and days 8–12-treated group) showed higher TEER values than the other groups (Additional file 1: Fig. S5). Expression of several tight junction markers, such as zonula occludens protein 1 (ZO-1), occludin, and claudin-5, revealed fragile and discontinuous tight junctions in controls (white arrows in Fig. 2d) and continuous tight junctions in the A-83-01-treated group (Fig. 2d). In addition, the mRNA expression level of matrix metallopeptidase 9 (MMP9), which increases BBB permeability, was significantly decreased in the A-83-01-treated group compared to controls (Additional file 1: Fig. S6).

To use iBMELCs in pharmacokinetic studies, stable maintenance of high-TEER values is essential. Thus, TEER values were measured every 6 h for approximately 2 weeks after seeding the cells on Transwell inserts. TEER values in the human brain have not been reported, unlike those reported in the rat brain [11]. Based on these values, we defined high-TEER as values ≥ 1000 Ω × cm^2^. In the A-83-01-treated group, high-TEER values were maintained for a longer period than in the control group in all cell lines (Fig. 2e). In addition, the positive cell ratio of caspase-3 to total number of cells was similar with or without A-83-01 at day 10, but significantly decreased in the A-83-01-treated group at day 19 (Additional file 1: Fig. S7).

### The effects of TGF-β inhibitor on the expression and activities of drug transporters

**Fig. 3:**
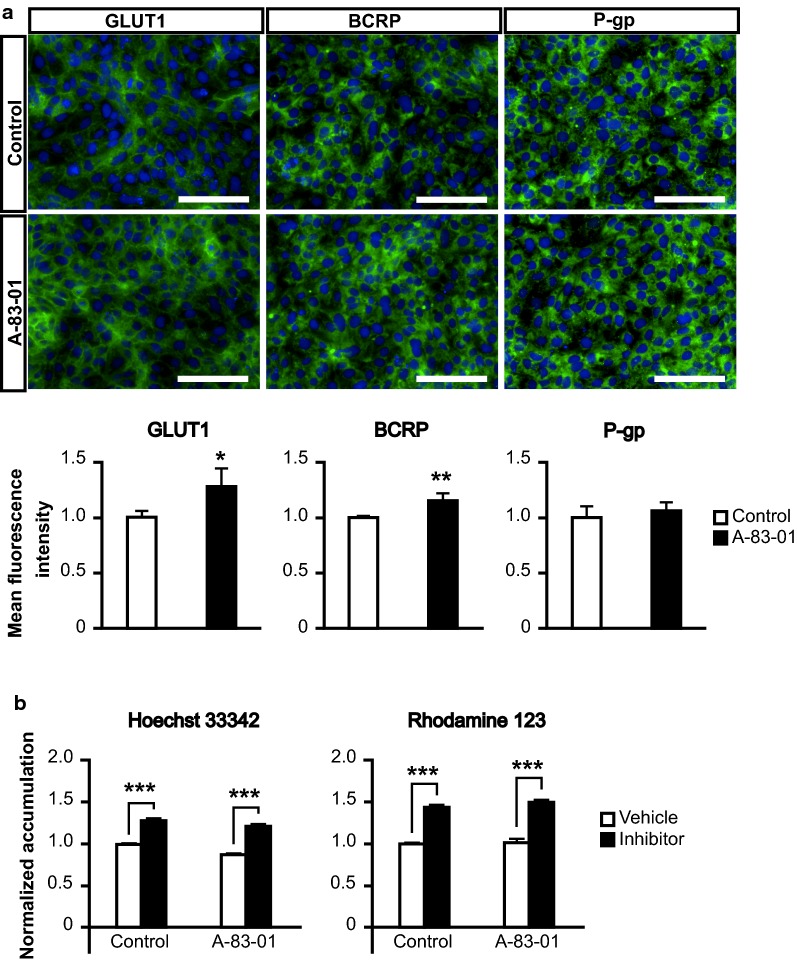
Protein expression and activities of efflux transporters in iBMELCs. **a** Immunofluorescence and mean fluorescence intensity of transporters (GLUT1, BCRP, and P-gp). Blue: DAPI. Scale bar, 100 μm. Statistical significance was calculated using the unpaired Student’s *t*-test (*p < 0.05, **p < 0.01), control = 1. Data are represented as the mean ± SD (*n* = 3). **b** Intercellular accumulation of each substrate of efflux transporters. iBMELCs were incubated with Hoechst 33342 (20 μM) or rhodamine 123 (10 μM) in the absence or presence of Ko143 (20 μM) or cyclosporin A (10 μM), respectively for 60 min at 37 °C. Relative fluorescence intensity values were normalized to protein content and self-normalized to the conditions without inhibitor. Data are represented as the mean ± SD (*n* = 3). Statistical significance was calculated using the unpaired Student’s *t*-test (***p < 0.001), vehicle of control = 1

Drug transporters play an important role in the selective transfer of substances to the brain. In the present study, we focused on the expression of the drug transporters GLUT1, BCRP, and P-gp because it was previously shown that these proteins are highly expressed in human BMECs in descending order of protein expression levels [32]. Addition of A-83-01 did not change the expression of P-gp, but significantly increased that of GLUT1 and BCRP compared to that in controls (Fig. 3a). To evaluate the activities, each substrate accumulation into iBMELCs was measured. The intercellular accumulation of BCRP or P-gp substrates was observed in both groups and the amounts were significantly increased by each inhibitor (Fig. 3b).

### Effects of TGF-β inhibitor on cryopreservation of iBMELCs

**Fig. 4:**
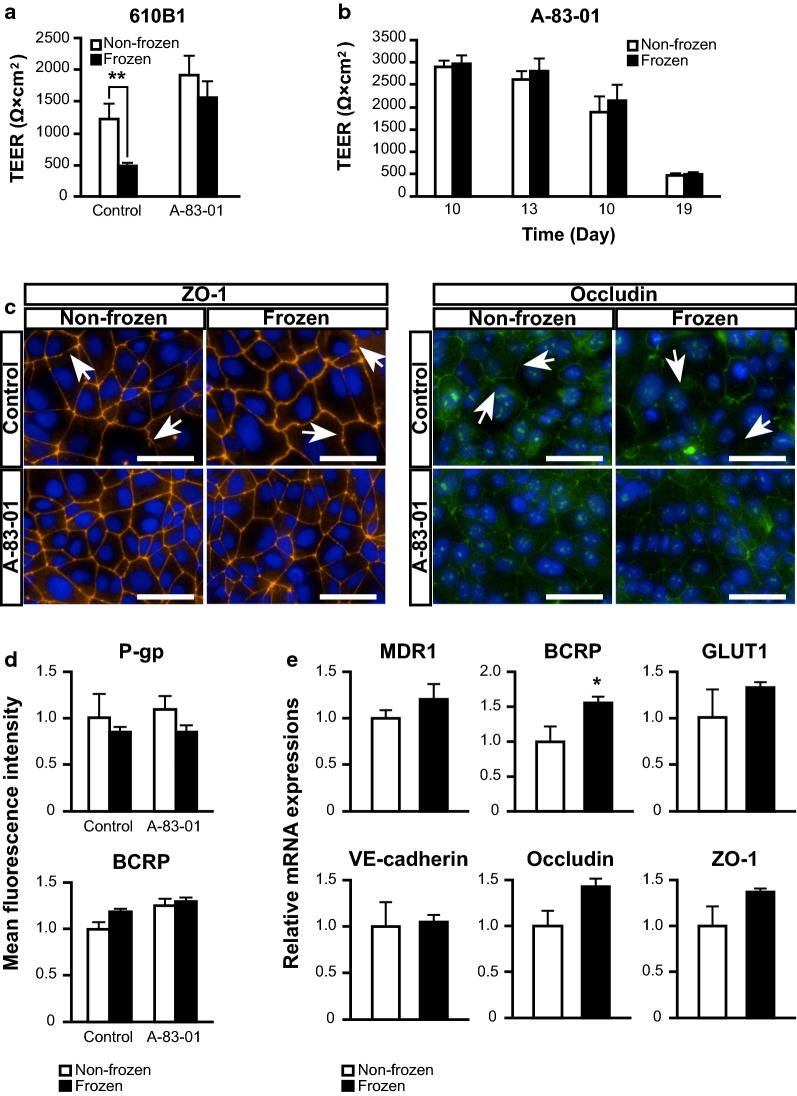
The effect of TGF-β inhibitor on freezing–thawing of BMECs. **a** Freezing–thawing was performed at day 8 and the TEER values were measured at day 10. Statistical significance was calculated using the unpaired Student’s *t*-test (**p < 0.01). Data are represented as the mean ± SD (*n* = 3). **b** Temporal changes in TEER values were observed in the BMECs with TGF-β inhibitor. Statistical significance was calculated using two-way repeated measures ANOVA. Data are represented as the mean ± SD (*n* = 3). **c** Immunofluorescence for the tight junction markers (ZO-1 and occludin). Blue: DAPI. White arrows: discontinuous tight junctions. Scale bar, 50 μm. **d** Mean fluorescence intensities of P-gp and BCRP staining were calculated. Statistical significance was calculated using the unpaired Student’s *t*-test, non-frozen of control = 1. Data are represented as the mean ± SD (*n* = 3). **e** Relative gene expression levels of *MDR1*, *BCRP*, *GLUT1*, *VE*-*cadherin*, *occludin*, and *ZO*-*1*. Statistical significance was calculated using the unpaired Student’s *t*-test (*p < 0.05), non-frozen = 1. Data are represented as the mean ± SD (*n* = 3)

Cell cryopreservation is required for establishing any process as a practical in vitro model. Therefore, the effect of freezing–thawing on iBMELC was investigated. iBMELCs were frozen on day 8, and the cells were thawed and seeded on Transwell inserts after 60–90 min or 1 month. TEER values on the second day after seeding were significantly decreased in frozen cells compared with non-frozen cells in the control group; however, the decrease was suppressed in the A-83-01-treated group (Fig. 4a, Additional file 1: Fig. S8). Furthermore, TEER values in the A-83-01-treated group were similar among two groups, frozen and non-frozen cells, between the second and eleventh day after seeding on the inserts (Fig. 4b). Immunofluorescence with tight junction markers showed that continuous tight junctions were observed in the A-83-01-treated group irrespective of freezing–thawing (Fig. 4c). The fluorescence intensity of P-gp and BCRP staining hardly changed between the non-frozen and frozen group in both the control and A-83-01-treated groups (Fig. 4d). The mRNA expression levels of *VE*-*cadherin*, *multiple drug resistance 1* (*MDR1*), *BCRP*, *GLUT1*, *occludin*, and *ZO*-*1* were also similar between the frozen and non-frozen group (Fig. 4e). Furthermore, TEER value was also increased by A-83-01 even in iBMELCs cryopreserved for 1 month. Furthermore, the TEER value in the A-83-01-treated group (approximately 2500 Ω × cm^2^) was equivalent to that of non-frozen cells (Additional file 1: Fig. S9).

## Discussion

Previous reports have shown that a co-culture system with BBB-related cells, such as pericytes, astrocytes, or neurons, enhances the barrier property of iBMELCs [15–21]. However, these systems require complex manipulation and high costs and are easily affected by lot-to-lot differences in co-cultured cells. Thus, these systems are useful models for the study of neuron toxicity, development of BBB, or diseases related to central nervous system; however, they are not suitable for predicting drug permeability using high throughput screening. Therefore, in this study, we focused on a differentiation method using small molecule compounds, which exhibit less lot-to-lot variability, high stability, and low-costs compared with co-culture systems or culture systems that use recombinant proteins. Here, we demonstrated that TGF-β signaling pathway inhibitor dramatically enhanced barrier integrity and endothelial characteristic of iBMELCs.

The TGF-β inhibitor we used remarkably increased the protein expression level of vascular endothelial cell marker VE-cadherin and the positive cell ratio of Ac-LDL uptake cells. These results suggest that TGF-β inhibition promotes differentiation of human iPSCs into BMELCs and increases proportion of endothelial cells among differentiated cells. These positive effects are reflected in our results by an enhancement in the ability of these cells to form blood vessel-like structures. In addition, iBMELCs showed significantly high-TEER values, low-permeability coefficient of Lucifer Yellow, and formed tighter continuous junctions when adding the TGF-β inhibitor compared to controls. TGF-β is known to mediate EndoMT [31], which increases BBB permeability [33]. Therefore, we hypothesized the enhancement of TEER occurred because of EndoMT suppression. However, TGF-β inhibitor hardly affected relatively mature endothelial cells, such as HUVECs and hCMEC/D3, in which EndoMT was previously observed [31, 34]. On the other hand, TGF-β inhibitor decreased the protein expression level of the mesenchymal cell marker, N-cadherin. Additionally, the TEER value was slightly increased by TGF-β inhibitor-treatment after the end of differentiation. Furthermore, iBMELCs treated with TGF-β inhibitor before the end of differentiation showed high-TEER values notably. Taken together, the increase of TEER might be caused not only by EndoMT suppression, but specifically by the promotive effect of TGF-β signaling pathway inhibition on iBMELCs during endothelial differentiation. Further, according to a previous study, retinoic acid-supplementation also enhances the protein expression level of VE-cadherin, which is a typical adherence junction maker and has barrier properties [16]. Considering that VE-cadherin is a positive regulator of tight junctions [35], it was suggested that an increase in the expression of VE-cadherin by supplementation of TGF-β inhibitor correlated with a significant increase in TEER value. Alternatively, it was also reported that TGF-β upregulates MMP9, which is a matrix metalloprotease that disrupts tight junctions, and increases the permeability of drugs in BBB [27]. In our study, the relative gene expression level of MMP9 was markedly decreased by the addition of TGF-β inhibitor. These results imply that suppression of MMP9 by the TGF-β inhibitor might contribute to robust tight junction formation. The fact that iBMELCs, treated with TGF-β inhibitor, maintained high-TEER value longer than the control group may also be involved in the suppression of MMP9. The effect of TGF-β inhibitor to maintain high-TEER value might be related to a TGF-β inhibitor apoptosis suppression, in accordance with the results of caspase-3 expression levels. From the results of efflux transport analysis, the activities could be evaluated in both control and A-83-01-treated groups. Although TGF-β inhibitor hardly affected activities of several drug transporters, it slightly increased the protein expression levels of BCRP and GLUT1. These results indicate that the characteristics of cells differentiated using TGF-β inhibitor became close to those of BMECs.

Our results suggested that the TGF-β inhibitor is a critical modulator of BMEC properties during differentiation to direct human iPSCs into BMELCs; it is also useful for the establishment of in vitro BBB models.

Cell cryopreservation is required for the application of a process to an in vitro model. The TEER values were significantly decreased in frozen cells in the control group. Wilson et al. have reported that cryopreservation impaired the barrier function of iBMELC [24]. Our results were consistent with this report. However, by adding the TGF-β inhibitor, we succeeded in maintaining robust tight junctions. Even when iBMELCs were cryopreserved for 1 month, high-TEER values could be maintained by the TGF-β inhibitor. Wilson et al. also reported that Y-27632 suppressed the decrease of TEER [24]. Thus, apoptosis inhibition may be one of the mechanisms that lead to strengthening of tight junctions. However, in the present study, the positive cell ratio of caspase-3 to total number of cells was not changed under non-frozen conditions, independently of whether we used or not A-83-01 at day 10. Thus, this phenomenon may be due to other effects of TGF-β inhibitors.

We confirmed the effect of TGF-β inhibition on iBMELCs differentiated by a specific protocol. However, there are several protocols, including Quian’s protocol [19] and Hollmann’s protocol [20], for the differentiation of iBMELCs. We speculate that TGF-β inhibitor also promotes the differentiation of iBMELCs in Hollmann’s protocol because the cells differentiated by Hollmann’s protocol are relatively similar to those differentiated by Lippmann’s protocol. In contrast, the characteristics of iBMELCs differentiated by Quian’s protocol are dissimilar to those differentiated by Lippmann’s protocol, and the effect of TGF-β inhibition in their protocol is not predictable. Therefore, future studies are warranted to address whether TGF-β inhibition can be applied to other protocols.

## Conclusions

In this study, we succeeded in significantly enhancing the function and endothelial characteristics of iBMELCs by adding a small molecular compound, a TGF-β inhibitor. Moreover, the iBMELCs could maintain high barrier function even after freezing–thawing. Therefore, we suggest that using a TGF-β inhibitor can help in developing high functional in vitro BBB models from iPSCs. Furthermore, iBMELCs differentiated using a TGF-β inhibitor can be potentially used as tools in pharmaceutical research in the future.

## Supplementary information

**Additional file 1: Fig. S1.** Endothelial characteristics of BMECs derived from multiple iPSC lines. **Fig. S2.** Protein expression levels of N-cadherin. **Fig. S3.** Phosphorylation levels of SMAD2. **Fig. S4.** Effect of TGF-β inhibitor on human immortalized BMEC (hCMEC/D3) and umbilical vein endothelial cells (HUVEC). **Fig. S5.** Effect of TGF-β inhibitor addition on TEER values before and after the end of differentiation. **Fig. S6.** Relative gene expression of MMP9 in A-83-01-treated cells. **Fig. S7**. Expression levels of caspase-3. **Fig. S8.** The effect of TGF-β inhibitor on freezing–thawing of BMECs derived from another iPSC line. **Fig. S9.** Effect of TGF-β inhibitor on the cryopreservation of iBMELCs. **Table S1.** PCR primer sequences. **Table S2.** Antibodies for immunofluorescence analysis. **Table S3.** Setting for the Opperetta High-Content Imaging System and minimum intensity of positive cells. **Table S4.** Antibodies for Western blotting analysis. Additional Experimental Procedures.

## Data Availability

The datasets used and/or analyzed in the current study are available from the corresponding author upon reasonable request.

## Abbreviations

Ac-LDL: Acetylated-low density lipoprotein
RA: All-trans retinoic acid
FGF2: Basic fibroblast growth factor
BSA: Bovine serum albumin
BCRP: Breast cancer resistant protein
DAPI: 4′,6-diamidino-2-phenylindole
EndoMT: Endothelial to mesenchymal transition
GLUT1: Glucose transporter 1
Matrigel: Growth factor reduced matrigel
HUVEC: Human umbilical vein endothelial cells
HPRT: Hypoxanthine phosphoribosyltransferase
iPSC: Induced pluripotent stem cells
iBMELCs: iPSC-derived BMECs
MMP9: Matrix metallopeptidase 9
MDR1: Multiple drug resistance 1
N-cadherin: Neural cadherin
PECAM1: Platelet endothelial cell adhesion molecule-1
P-gp: P-glycoprotein
PBS: Phosphate buffered saline
PDS: Platelet-poor plasma derived bovine serum
SDS-PAGE: Sodium dodecyl sulfate–polyacrylamide gel electrophoresis
TEER: Transendothelial electrical resistance
TGF-β: Transforming growth factor beta
UM: Unconditioned medium
VE-cadherin: Vascular endothelial cadherin
vWF: Von Willebrand factor
ZO-1: Zonula occludens protein 1
