# Supplementary material for: Inhibition of transforming growth factor beta signaling pathway promotes differentiation of human induced pluripotent stem cell-derived brain microvascular endothelial-like cells
Source: Fluids Barriers CNS. 2020 May 26;17:36. doi: 10.1186/s12987-020-00197-1 (PMC7249446; doi:10.1186/s12987-020-00197-1)
Supplement: Supplementary file 1 — Additional file 1: Fig. S1. Endothelial characteristics of BMECs derived from multiple iPSC lines. Fig. S2. Protein expression levels of N-cadherin. Fig. S3. Phosphorylation levels of SMAD2. Fig. S4. Effect of TGF-β inhibitor on human immortalized BMEC (hCMEC/D3) and umbilical vein endothelial cells (HUVEC). Fig. S5. Effect of TGF-β inhibitor addition on TEER values before and after the end of differentiation. Fig. S6. Relative gene expression of MMP9 in A-83-01-treated cells. Fig. S7. Expression levels of caspase-3. Fig. S8. The effect of TGF-β inhibitor on freezing–thawing of BMECs derived from another iPSC line. Fig. S9. Effect of TGF-β inhibitor on the cryopreservation of iBMELCs. Table S1. PCR primer sequences. Table S2. Antibodies for immunofluorescence analysis. Table S3. Setting for the Opperetta High-Content Imaging System and minimum intensity of positive cells. Table S4. Antibodies for Western blotting analysis. Additional Experimental Procedures. [file 12987_2020_197_MOESM1_ESM.pdf]

ADDITIONAL FILE 1

Inhibition of transforming growth factor beta signaling pathway promotes differentiation of human induced pluripotent stem cell-derived brain microvascular endothelial-like cells

Misaki Yamashita, Hiromasa Aoki, Tadahiro Hashita, Takahiro Iwao, Tamihide Matsunaga*

Department of Clinical Pharmacy, Graduate School of Pharmaceutical Sciences, Nagoya City University, Nagoya, Japan

E-mail addresses

Misaki Yamashita; y.misaki47@gmail.com

Hiromasa Aoki; aokihiromasa812@gmail.com

Tadahiro Hashita; thashita@phar.nagoya-cu.ac.jp

Takahiro Iwao; tiwao@phar.nagoya-cu.ac.jp

Tamihide Matsunaga; tmatsu@phar.nagoya-cu.ac.jp

***Corresponding author:** Tamihide Matsunaga, Ph.D.

Department of Clinical Pharmacy, Graduate School of Pharmaceutical Sciences, Nagoya City University, 3-1 Tanabe-dori, Mizuho-ku, Nagoya 467-8603, Japan

Tel: +81-52-836-3751

Fax: +81-52-836-3751

E-mail: tmatsu@phar.nagoya-cu.ac.jp

Additional Figures

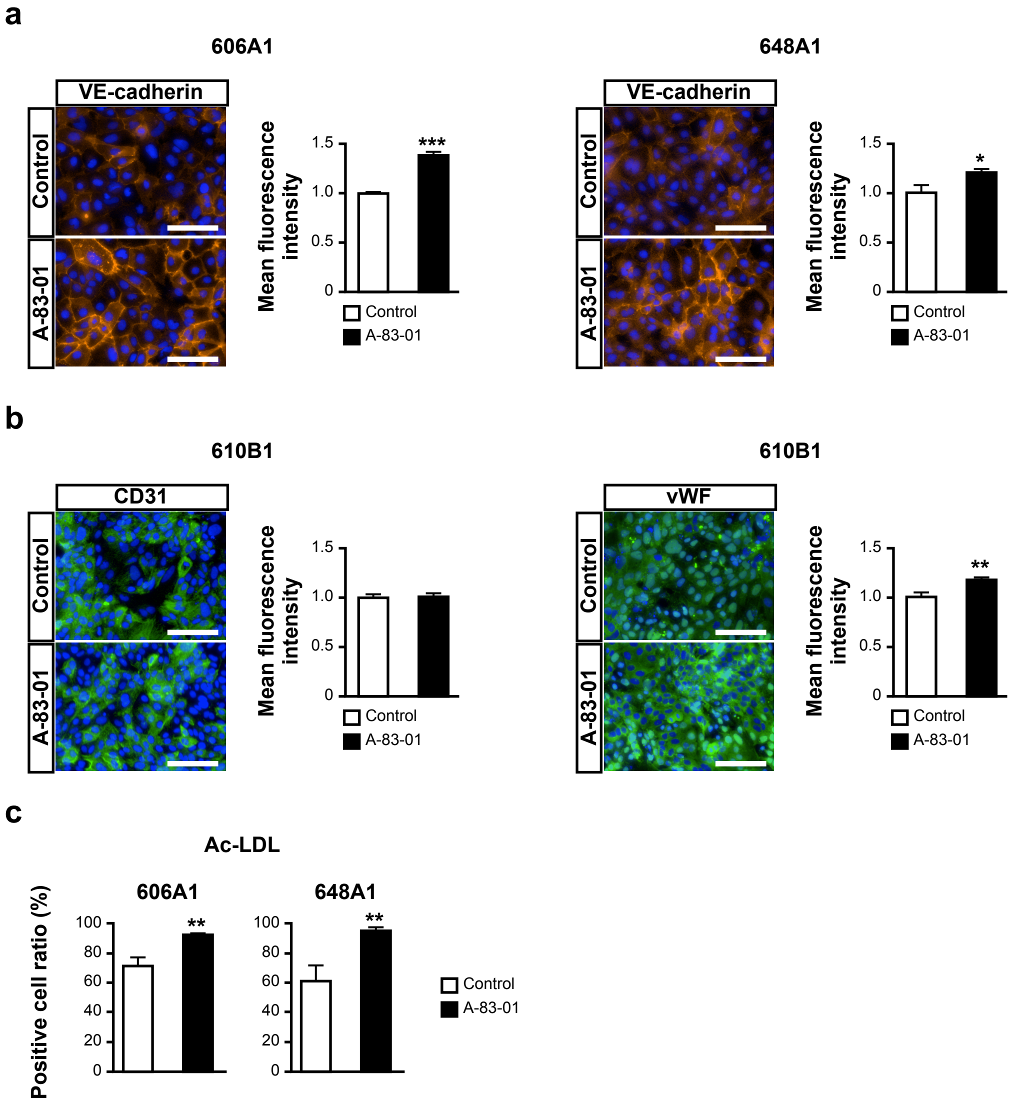

Fig. S1. Endothelial characteristics of BMECs derived from multiple iPSC lines

(a) Immunofluorescence for the endothelial cell adhesion molecule (VE-cadherin; red) of BMECs derived from multiple iPSC lines. Blue: DAPI. Scale bar, 100 μm. Statistical significance was calculated using the unpaired Student's *t*-test (*$p < 0.05$, ***$p < 0.001$), control = 1. Data are represented as the mean ± SD (n = 3).

(b) Immunofluorescence for the endothelial cell markers (CD31 and vWF; green) of 610B1 derived BMECs. Blue: DAPI. Scale bar, 100 μm. Statistical significance was calculated using the unpaired Student's *t*-test (**$p < 0.01$), control = 1. Data are represented as the mean ± SD (n = 3).

(c) Ac-LDL (red) uptake assay. Blue: Hoechst 33342. Scale bar, 100 μm. Positive cell ratio was defined as the number of cells with Ac-LDL uptake to the total number of cells. Statistical significance was

calculated using the unpaired Student's t-test (*** $p < 0.001$). Data are represented as the mean ± SD (n = 3).

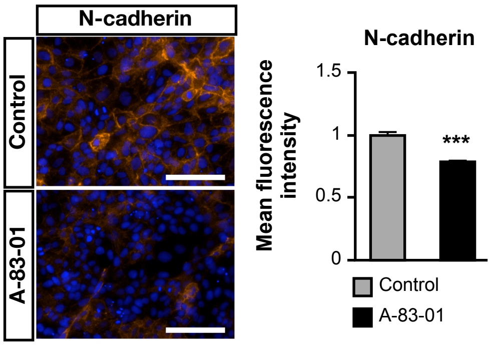

Fig. S2. Protein expression levels of N-cadherin

Immunofluorescence with mesenchymal cell adhesion molecule (N-cadherin; red). Blue: DAPI. Scale bar, 100 μm. Fluorescence intensity was calculated as the average fluorescence intensity of 6 fields/well in three wells (n = 3) using Harmony software. The mean fluorescence intensity (control = 1) was calculated by dividing the fluorescence intensity by the total cell number. Statistical significance was calculated using the unpaired Student's t-test (*** $p < 0.001$). Data are represented as the mean ± SD (n = 3).

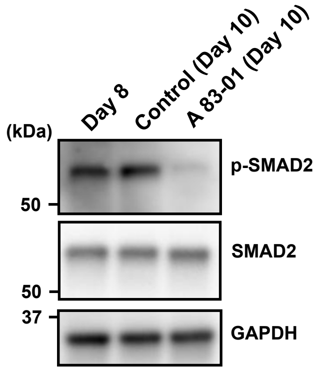

Fig. S3. Phosphorylation levels of SMAD2

The phosphorylation levels of SMAD2 in iBMELCs were detected by Western blotting analysis.

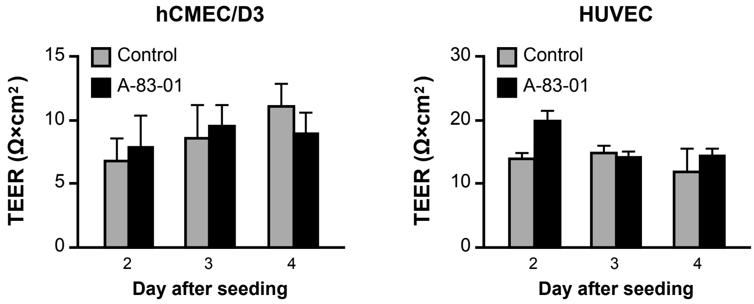

Fig. S4. Effect of TGF-β inhibitor on human immortalized BMEC (hCMEC/D3) and umbilical vein endothelial cells (HUVEC)

Statistical significance was calculated using two-way repeated measures ANOVA. Data are represented as the mean ± SD (hCMEC/D3, $n = 4$; HUVEC, $n = 3$).

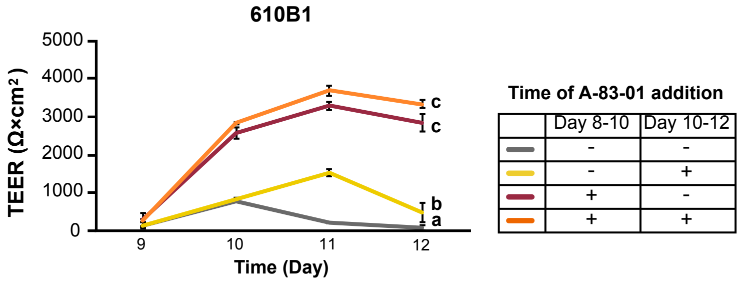

Fig. S5. Effect of TGF-β inhibitor addition on TEER values before and after the end of differentiation

Statistical significance was calculated using two-way repeated measures ANOVA. Data are represented as the mean ± SD ($n = 3$), with different letters (a, b, and c) including significantly different ($p < 0.05$) values.

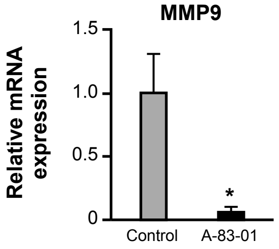

Fig. S6. Relative gene expression of *MMP9* in A-83-01-treated cells

Statistical significance was calculated using the unpaired Student's t-test ($*p < 0.05$), control = 1. Data are

represented as the mean ± SD ($n = 3$).

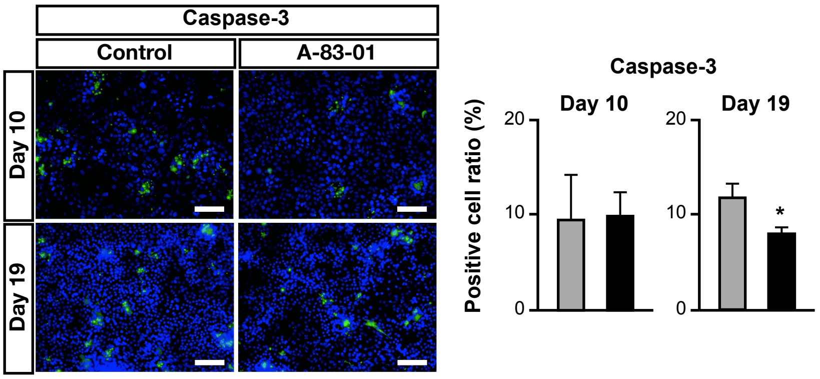

Fig. S7. Expression levels of caspase-3

Immunofluorescence for the apoptosis marker caspase-3 (green). Blue: DAPI. Scale bar, 100 µm. Positive cells were counted as the average number of 6 fields/well in three wells ($n = 3$) using the Operetta High-Content Imaging System. The positive cell ratio was calculated by dividing the number of caspase-positive cells by the total cell number. Statistical significance was calculated using the unpaired Student's *t*-test (*$p < 0.05$). Data are represented as the mean ± SD ($n = 3$).

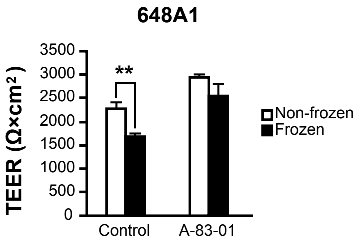

Fig. S8. The effect of TGF-β inhibitor on freezing–thawing of BMECs derived from another iPSC line

Freezing–thawing was performed at day 8 and the TEER values were measured at day 10. Statistical significance was calculated using the unpaired Student's *t*-test (**$p < 0.01$). Data are represented as the mean ± SD ($n = 3$).

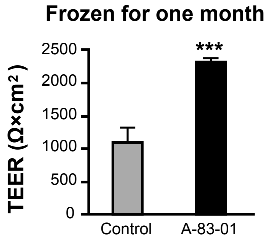

Fig. S9. Effect of TGF-β inhibitor on the cryopreservation of iBMELCs

Statistical significance was calculated using the unpaired Student's *t*-test (***$p < 0.001$). Data are represented as the mean ± SD ($n = 3$).

Additional Tables

Table S1. PCR primer sequences

Genes	Forward primer sequence (5′ → 3′)	Reverse primer sequence (5′ → 3′)
VE-cadherin	GATTTGGAACCAGATGCACA	ACTTGGCATTCTTGCGACTC
MDR1	CCCATCATTGCAATAGCAGG	TGTTCAAACTTCTGCTCCTGA
BCRP	AGATGGGTTTCCAAGCGTTCAT	CCAGTCCCAGTACGACTGTGACA
GLUT1	GAAGAGAGTCGGCAGATGATG	GGAGTAATAGAAGACAGCGTTGATG
Occludin	TCCAATGGCAAAGTGAATGA	GCAGGTGCTCTTTTTGAAGG
ZO-1	CGAGGGATAGAAGTGCAAGTAGA	TATTCTTCATTTTTCCGGGATTT
MMP9	TGTATTTGTTCAAGGATGGGAAG	AGAAGAAAAGCTTCTTGGAGAGC
HPRT	CTTTGCTTTCCTTGGTCAGG	TCAAGGGCATATCCTACAACA

Table S2. Antibodies for immunofluorescence analysis

Targets	Manufacturer	Catalogue number	Species	Dilution
VE-cadherin	Santa Cruz	sc-9989	Mouse	1:25
CD31	Abcam	ab28364	Rabbit	1:25
vWF	Abcam	ab6994	Rabbit	1:100
P-gp	Abcam	ab10333	Mouse	1:25
BCRP	Abcam	ab3380	Mouse	1:50

GLUT1	Thermo	MA5-11315	Mouse	1:50
Occludin	Thermo	71-1500	Rabbit	1:50
ZO-1	Thermo	33-9100	Mouse	1:100
Claudin-5	Thermo	35-2500	Mouse	1:25
N-cadherin	BD Biosciences	610920	Mouse	1:25
Caspase-3	CST	9661S	Rabbit	1:100
Alexaflour 488 anti-rabbit	Thermo	A-21206	Donkey	1:200
Alexafluor 568 anti-mouse	Thermo	A-11004	Goat	1:200

Table S3. Setting for the Opperetta High-Content Imaging System and minimum intensity of positive cells

Settings	Conditions
Plate type	PerkinElmer Cell Carrier 96
Objective	20× long WD
Opt. mode	Non-confocal
Excitation	100%
Calculate program (A)	Intensity Cytoplasm Alexa 488, 568 Mean
Calculate program (B)	Sum of Intensity Cytoplasm Alexa 488, 568 Mean / Number of Objects

Figs. 1c, S1c, and S7 were calculated using program (A). Figs. 1b, 3a, 4d, S1a, S1b, and S2 were calculated using program (B).

Target	Ac-LDL	Caspase-3
Fig. 1c	230	
Fig. S1c (606A1)	310	
Fig. S1c (648A1)	410	
Fig. S7 (Day 10)		108
Fig. S7 (Day 19)		750

Table S4. Antibodies for Western blotting analysis

Targets	Manufacturer	Catalogue number	Species	Dilution
GAPDH (HRP)	Wako	35-2500	Mouse	1:1000
SMAD2	CST	5339	Rabbit	1:1000
p-SMAD2 (Ser465/467)	CST	3108	Rabbit	1:100
Rabbit (HRP)	CST	7074	Goat	1:5000

Additional Experimental Procedures

Culture of hCMEC/D3

The hCMEC/D3 BBB cell line was purchased from Merck Millipore (Burlington, MA, USA) and cultured in hCMEC/D3 medium: Endothelial Cell Basal Medium-2 (EBM-2) supplemented with 5% fetal bovine serum (Sigma-Aldrich), 5 µg/mL ascorbic acid, 1% chemically defined lipid concentrate, 10 µM HEPES solution (Sigma-Aldrich), 1% penicillin-streptomycin solution (Biological Industries, Cromwell, CT, USA), 1.4 µM hydrocortisone (Wako) and 1 ng/mL FGF2. The cells were dissociated with TrypLE select and plated at a 1:5-split ratio. Before the TEER measurement assay, cells were seeded onto 0.3-cm^2 Transwell-Clear permeable inserts (0.4 µm pore size) coated with collagen I at a density of 5.0 × 10^4 cells/cm^2 and cultured in hCMEC/D3 medium with or without TGF-β inhibitor (1 µM). TEER values were measured starting 2 days after seeding.

Culture of human umbilical vein endothelial cells

HUVECs were purchased from ScienCell and cultured in Endothelial Cell Media (ScienCell) according to the manufacturer's instructions. The cells were dissociated with TrypLE select and seeded onto 0.3-cm^2 Transwell-Clear permeable inserts (0.4 µm pore size) coated with 20 µg/mL fibronectin. The cells were seeded at a density of 4.0 × 10^4 cells/cm^2 and cultured in EC medium lacking RA with or without TGF-β inhibitor (1 µM) for 24 hours. TEER values were measured starting from 2 days after seeding.
